# An anti-ErbB2 fully human antibody circumvents trastuzumab resistance

**DOI:** 10.18632/oncotarget.11562

**Published:** 2016-08-24

**Authors:** Qiong Lu, Lingfei Wang, Yajun Zhang, Xiaojie Yu, Chao Wang, Huajing Wang, Yang Yang, Xiaodan Chong, Tian Xia, Yanchun Meng, Yuxiao Wang, Cuihua Lu, Lijun Zhou, Bohua Li

**Affiliations:** ^1^ International Joint Cancer Institute, The Second Military Medical University, Shanghai, People's Republic of China; ^2^ School of Medicine, Nankai University, Tianjin, People's Republic of China; ^3^ Department of Gastroenterology, The Affiliated Hospital of Nantong University, Nantong, Jiangsu, People's Republic of China; ^4^ Central Laboratory, Navy General Hospital, Beijing People's Republic of China

**Keywords:** ErbB2, programmed cell death, trastuzumab resistance, domain I- specific antibody, breast cancer

## Abstract

Trastuzumab, an anti-HER2/ErbB2 humanized antibody, has shown great clinical benefits in ErbB2-positive breast cancer treatment. Despite of its effectiveness, response rate to trastuzumab is limited and resistance is common. Here, we developed a new anti-ErbB2 antibody, denoted as H2-18, which was isolated from a phage display human antibody library. Previous studies have demonstrated that trastuzumab recognizes the juxtamembrane region of domain IV, and pertuzumab, another humanized ErbB2-specific antibody, binds to ErbB2 near the center of domain II. Our crystallographic analysis showed that the epitope recognized by H2-18 is within domain I of the ErbB2 molecule. H2-18 potently induced programmed cell death (PCD) in both trastuzumab-sensitive and -resistant breast cancer cell lines, while trastuzumab and pertuzumab, either used alone or in combination, only exhibits very weak PCD-inducing activity. More importantly, H2-18 could inhibit the growth of trastuzumab-resistant breast cancer cells far more effectively than trastuzumab plus pertuzumab, both *in vitro* and *in vivo*. In conclusion, H2-18 shows a unique ability to overcome trastuzumab resistance, suggesting that it has the great potential to be translated to the clinic.

## INTRODUCTION

Breast cancer becomes the most lethal malignancy in women world-wide in recent years, and its incidence and mortality rate are still climbing high in both developed and developing countries. Overexpression of ErbB2 is found in 25%-30% of human breast cancers [1, 2] and is associated with tumor progression and poor prognosis [3]. ErbB2 (HER2) is a member of the epidermal growth factor receptor family, which involves critical signaling functions in breast tissues. Because ErbB2 lacks ligand-binding activity, its activation is relied on the formation of homodimers and/or heterodimers with other ligand-activated partners (HER1, 3 and 4) [4–6]. The dimers stimulate ErbB2 phosphorylation, initiate downstream signaling events including PI3K/AKT and MAPK/ERK pathways, and culminate in tumor growth [4–6].

Trastuzumab (Herceptin) is a humanized monoclonal antibody directed against the extracellular domain IV of ErbB2. It is the first anti-ErbB2 antibody approved for clinical use for ErbB2-amplified metastatic breast cancer by FDA in 1998 [7] and has been the standard therapy till now. The possible mechanisms involving its action include ErbB2 downregulation and endocytosis, disruption of ErbB2-ErbB3 dimers followed by inhibition of PI3K/AKT pathway, cell-cycle arrest and antibody-dependent, cell-mediated cytotoxicity (ADCC) [8].

However, clinical data show that about 70% of the patients with ErbB2-overexpressing breast cancer do not respond to trastuzumab treatment. Many studies attempt to elucidate the mechanisms underlying trastuzumab resistance. One major mechanism involves aberrant activation of PI3K/AKT signaling. Deficiency of the tumor suppressor phosphatase PTEN or mutation in PIK3CA leads to persistent activation of PI3K/AKT signaling [9–14]. Extensive researches propose strategies to abrogate or delay trastuzumab resistance. For example, combination trastuzumab with tyrosine kinase inhibitors or heat shock protein inhibitors, concomitant treatment with antiestrogen therapies or blockade of other pathways, development of an antibody-drug conjugate (trastuzumab-DM1), and *etc*.[15, 16]. Among these, one strategy is addition of pertuzumab to trastuzumab. Many studies have further shown that even when cancers progress after trastuzumab therapy, ErbB2 still remains a valid therapeutic target [17–20], suggesting that ErbB2 still represents a major vulnerability for ErbB2-positive cancer cells [21]. Different from trastuzumab that inhibited ErbB2 homodimer and ligand-independent heterodimer, pertuzumab, another therapeutic antibody directed against domain II of ErbB2, efficiently inhibited ErbB2-ErbB3 complex formation when cells were stimulated with ErbB3 ligand. The combination of these two antibodies exhibits robust clinical success. Nevertheless, the objective response rate is only 24.2% and complete response only around 8% [18]. Thus, there is an urgent need to improve ErbB2-directed therapy. Here, we report a novel anti-ErbB2 fully human monoclonal antibody H2-18, which binds to domain I of ErbB2 and induces programmed cell death (PCD). More importantly, in trastuzumab-resistant breast cancer cell lines, it exhibits more potent antitumor efficacy than trastuzumab in combination with pertuzumab.

## RESULTS

### Construction and expression of H2-18

ErbB2-specific single chain antibody (H2-18-scFv) was selected from a large phage display antibody library [22] and then converted to complete (IgG1, κ) antibody (H2-18). H2-18 was constructed, expressed and purified using a similar method as described in our previous report [21]. Briefly, the heavy-chain and light chain expression vectors were co-transfected into CHO-K1 cells. After transfection, the stable transfectants were isolated by limiting dilution in the presence of G418 (500 mg/mL). The cell clone producing the highest amount of antibodies was grown in serum-free medium. Finally, H2-18 was purified by affinity chromatography on Protein A Sepharose (GE Healthcare).

It has been reported that trastuzumab binds to the juxtamembrane region of domain IV of ErbB2, and pertuzumab recognizes domain II. Recently, we have determined the crystal structure of ErbB2 in complex with H2-18. Our crystallographic analysis confirmed that the epitope of H2-18 is in domain I of ErbB2 [23]. Competitive binding assays also showed that H2-18 did not compete with either trastuzumab or pertuzumab for binding to ErbB2 (Supplementary Figure S1).

### H2-18 suppresses the *in vitro* proliferation of trastuzumab-resistant cell lines

**Figure 1:**
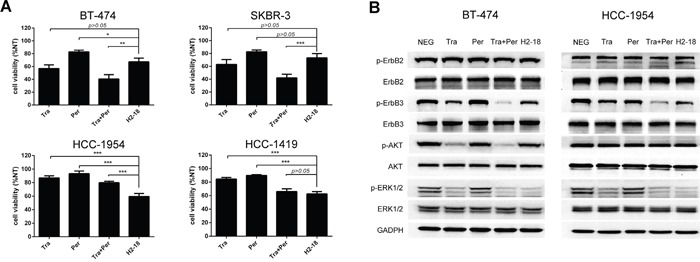
The antiproliferative activity of H2-18 in ErbB2-overexpressing breast cancer cell lines **A.** MTS assay assessing the proliferation of BT-474, SKBR-3, HCC-1954 and HCC-1419 cells upon treatment with control IgG, trastuzumab, pertuzumab, trastuzumab plus pertuzumab, and H2-18. All the cells were incubated with 10 μg/ml indicated anti-ErbB2 antibodies for 5 days. Results are shown as percentage of control cell proliferation. Every experiment was repeated 3 times. Error bars, SD. *, *P*<0.05; **, *P*<0.01; ***, *P*<0.001; ANOVA. **B.** Immunoblots evaluating the cell signaling changes of 10 μg/ml of control IgG, trastuzumab, pertuzumab, trastuzumab plus pertuzumab, and H2-18. Every experiment was repeated 3 times.

In trastuzumab-sensitive SKBR-3 and BT-474 cell lines, the growth inhibition activity of H2-18 was weaker than trastuzumab alone and trastuzumab plus pertuzumab (Figure 1A). However, in trastuzumab-resistant cell line HCC-1954, H2-18 inhibited the cell proliferation more effectively than did trastuzumab, pertuzumab, and trastuzumab plus pertuzumab (Figure 1A). As shown in Figure 1A, the inhibition of proliferation caused by both trastuzumab and pertuzumab was less than 20% in HCC-1954 cells. Even when trastuzumab and pertuzumab were used in combination, the growth inhibition rate was only 30% (Figure 1A). Strikingly, H2-18 could decrease the cell viability by 40-50% (Figure 1A).

### H2-18 significantly inhibits MAPK/ERK pathway but not PI3K/AKT pathway in trastuzumab-resistant cell lines

To examine the effect of H2-18 on ErbB2 signaling pathway, the trastuzumab-sensitive cell line BT-474 and the trastuzumab-resistant cell line HCC-1954 were treated with 5μg/ml anti-ErbB2 antibodies for 4h, and then cell lysates were subjected to western blot. In both BT-474 and HCC-1954 cell lines, no significant difference in pErbB2 was detected between the cells treated with indicated mAbs and that with control IgG (Figure 1B). ErbB3 phosphorylation was clearly reduced when cells were treated with trastuzumab (Figure 1B). The addition of pertuzumab to trastuzumab further reduced ErbB3 phosphorylation (Figure 1B). And in both cell lines, H2-18 inhibited ErbB3 phosphorylation as effectively as trastuzumab (Figure 1B).

Next, we investigated the changes in two downstream pathways of active ErbB2: MAPK/ERK and PI3K/AKT signaling. In both BT-474 and HCC-1954 cell lines, trastuzumab was more effective than pertuzumab in decreasing ERK1/2 phosphorylation (Figure 1B). Compared with either mAb alone, the combination of trastuzumab and pertuzumab caused a marked decrease in the level of pERK1/2 (Figure 1B). H2-18 inhibited ERK1/2 phosphorylation similarly to trastuzumab plus pertuzumab in both BT-474 and HCC-1954 cell lines (Figure 1B).

In BT-474 cell line, trastuzumab substantially reduced Akt phosphorylation (Figure 1B). The addition of pertuzumab to trastuzumab resulted in a more significant decrease in phospho-Akt compared with trastuzumab alone (Figure 1B). H2-18 did not reduce pAkt obviously (Figure 1B). In HCC-1954 cell line, however, no significant decrease in pAkt was induced by trastuzumab, pertuzumab, trastuzumab plus pertuzumab, or H2-18 (Figure 1B).

### H2-18 potently induces apoptosis in ErbB2-overexpressing breast cancer cell lines

**Figure 2:**
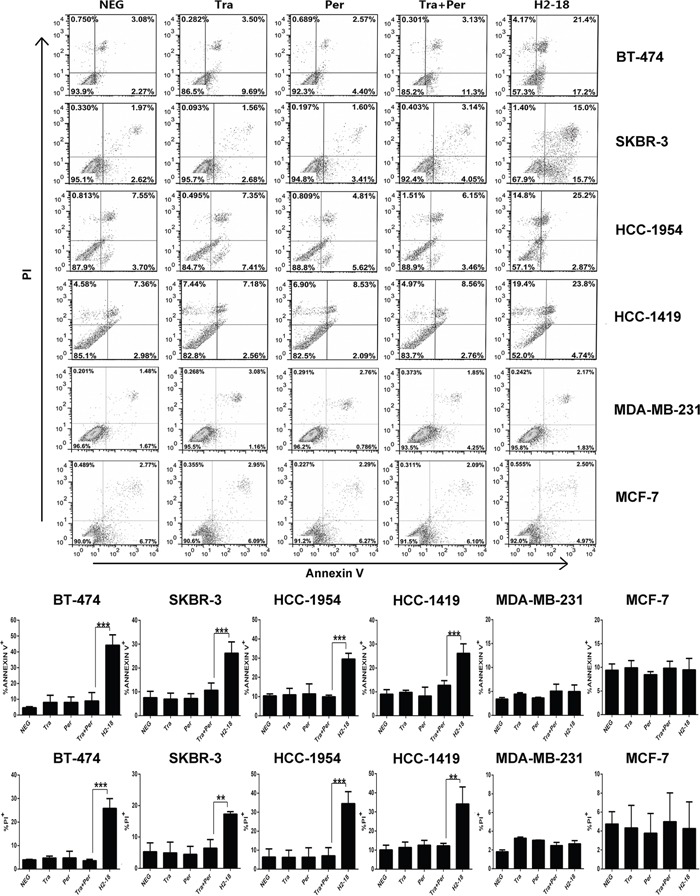
H2-18 potently induces apoptosis in ErbB2-overexpressing breast cancer cell lines Cell death induced by control IgG, trastuzumab, pertuzumab, trastuzumab plus pertuzumab, and H2-18 in high-ErbB2-expressing (BT-474, SKBR-3, HCC-1954, HCC-1419) and low-ErbB2-expressing (MDA-MB-231, MCF-7) was measured by flow cytometry using Annexin V/PI detecting kit. Every experiment was repeated 3 times. Error bars, SD. *, *P*<0.05; **, *P*<0.01; ***, *P*<0.001; ANOVA.

We used flow cytometry to determine the apoptosis-inducing activity of H2-18 in BT-474, SKBR-3, HCC-1954, HCC-1419 cell lines by using Dead Cell Apoptosis Kit. In H2-18-treated HCC-1954 cells, the percentage of Annexin V-positive cells is 28.07%, far higher than that of HCC-1954 cells treated with trastuzumab and pertuzumab, either alone or in combination (Figure 2). Similarly, H2-18 could induce much more PI-positive HCC-1954 cells than did all the other mAbs (Figure 2). Similar results were observed with BT-474, SKBR-3, and HCC-1419 cell lines (Figure 2). BT-474, SKBR-3, HCC-1419 and HCC-1954 are all ErbB2-overexpressing breast cell lines (Supplementary Figure S2). Next, we investigated the apoptosis-inducing activity of H2-18 in MDA-MB-231 or MCF-7 cell lines, which express very low levels of ErbB2 (Supplementary Figure S2). Our data showed that all the anti-ErbB2 antibodies, including H2-18, could not effectively trigger apoptosis in both cell lines (Figure 2), suggesting that the apoptosis-inducing activity of H2-18 is ErbB2-specific.

### Cell death induced by H2-18 is caspase- and autophagy-independent

**Figure 3:**
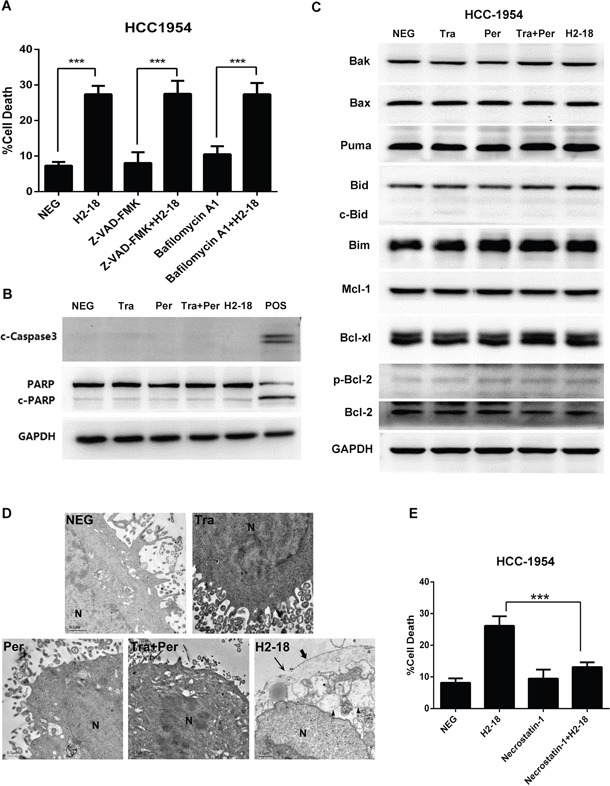
H2-18-induced cell death is RIP1-dependent programmed necrosis **A.** The effect of pan caspase inhibitor Z-VAD-FMK and autophagy inhibitor bafilomycin A1 on H2-18-induced cell death in HCC-1954 cells. Data are shown as means ± SD. *, *P*<0.05; **, *P*<0.01; ***, *P*<0.001; Student's unpaired *t* test. **B.** Immunoblots assessing the key components in caspase signaling in HCC-1954 cells treated with control IgG, trastuzumab, pertuzumab, pertuzumab plus trastuzumab, and H2-18. Lysates of HCC-1954 cell treated with NaN3 were used as positive control. **C.** Immunoblots examining Bcl-2 family in HCC-1954 cells treated with control IgG, trastuzumab, pertuzumab, pertuzumab plus trastuzumab, and H2-18. **D.** TEM images of HCC-1954 cells treated with control IgG, trastuzumab, pertuzumab, pertuzumab plus trastuzumab, and H2-18 for 48 hours. Representative images are shown. H2-18-treated HCC-1954 cells exhibited shedding of villi(➨), disruption of the plasma membrane(→), extensive cytoplasmic vacillation (▲), and intact nuclei (N). **E.** The effect of Nec-1 on H2-18-induced cell death in HCC-1954 cells. Every experiment was repeated 3 times. Data are shown as means ± SD. *, *P*<0.05; **, *P*<0.01; ***, *P*<0.001; ANOVA.

To determine whether caspase and autophagy pathways were involved in H2-18-induced cell death, the cell-permeant pan-caspase inhibitor Z-VAD-FMK and the autophagy inhibitor bafilomycin A1 were used to treat the HCC-1954 cell line. The results showed that both Z-VAD-FMK and bafilomycin A1 had no detectable effect on H2-18-induced cell death (Figure 3A). Accordingly, the results from western blotting indicated that no cleaved caspases 3 was observed in H2-18-treated HCC-1954 cells (Figure 3B, Supplementary Figure S4). No cleavage of PARP, a well-known substrate of activated caspases, was observed (Figure 3B). Compared with the negative control, H2-18 did not induce any significant change in LC3 proteins (Supplementary Figure S5). H2-18 also failed to change the expression of pro-apoptotic proteins (Bak, Bax, Puma, Bid, Bim) and pro-survival proteins (Mcl-1, Bcl-xl, p-Bcl-2, Bcl-2) (Figure 3C), suggesting that Bcl-2 family were not involved in H2-18-induced cell death.

### H2-18-induced cell death is RIP1-dependent

Transmission electron microscope (TEM) was performed to investigate the morphological changes of cells treated with H2-18. As shown in Figure 3D, the classic apoptotic changes were absent in the H2-18-treated cells, such as chromatin condensation, nuclear fragmentation, cell shrinkage, cytoskeletal disruption and formation of apoptotic body, suggesting that these cells did not undergo apoptosis. On the other hand, extensive cytoplasmic vacillation was observed in H2-18 treated cells (Figure 3D). Besides, H2-18-treated cells also possessed other features of necrosis, including shedding of villi, disruption of the plasma membrane and intact nuclei (Figure 3D). Furthermore, previous studies have found that not only apoptotic but also caspase-independent necrotic cells showed Annexin V-positive/PI-negative staining before they became PI-positive [24]. As mentioned above, H2-18 treatment led to an increase in the proportion of both PI-positive and Annexin V-positive/PI-negative cells, which could not exclude the possibility of necrosis. Hence, we hypothesized that H2-18 may trigger programmed necrosis. To our knowledge, unlike classic necrosis, programmed necrosis is a unique type of cell death that is controllable and is associated with specific signaling events. And RIP1 (receptor-interacting protein 1) is the key node of programmed necrosis [25]. It is well known that RIP1 is the only active site of necrostatin-1 (Nec-1), which can specifically bind the serine/threonine kinase of RIP1 and inhibit kinase activity [26]. When HCC-1954 cells were treated with H2-18 in the presence of Nec-1, Nec-1 could effectively abrogate the ability of H2-18 to trigger cell death (Figure 3E).

### JNK pathway and ROS production may participate in H2-18-induced programmed cell death

**Figure 4:**
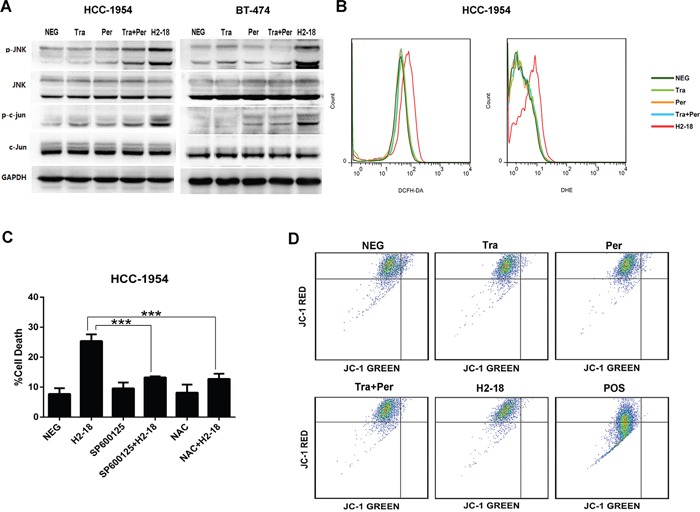
ROS and JNK take part in H2-18-induced programmed cell death **A.** Immunoblots examining the phosphorylation of JNK and c-Jun in HCC-1954 and BT-474 cells treated with control IgG, trastuzumab, pertuzumab, pertuzumab plus trastuzumab, and H2-18. **B.** DCFH-DA or DHE was used to determine the ROS production in HCC-1954 cells treated with control IgG, trastuzumab, pertuzumab, pertuzumab plus trastuzumab, and H2-18. **C.** The effect of JNK inhibitor SP600125 or ROS scavenger NAC on H2-18-induced cell death in HCC-1954 cells. Data are shown as means ± SD. *, *P*<0.05; **, *P*<0.01; ***, *P*<0.001; ANOVA. **D.** JC-1 staining was used to measure the mitochondrial membrane potential in HCC-1954 cells treated with control IgG, trastuzumab, pertuzumab, pertuzumab plus trastuzumab, and H2-18. HCC-1954 cells treated with CCCP was used as positive control. Every experiment was repeated 3 times.

As c-Jun N-terminal kinase (JNK) pathway and reactive oxygen species (ROS) production were often involved in RIP1-dependent programmed necrosis [27, 28], we investigated the changes of pJNK and p-c-Jun by western blotting and measured the production of ROS including hyperoxide and superoxide anion by flow cytometry. In both HCC-1954 and BT-474 cell lines, H2-18 increased pJNK and p-c-Jun to greater extent than did pertuzumab, trastuzumab or pertuzumab plus trastuzumab (Figure 4A). And excessive ROS production in HCC-1954 cells was triggered only by H2-18, but not by the other anti-ErbB2 mAbs (Figure 4B, Supplementary Figure S3).

To confirm that the generation of ROS and activation of JNK correlated with H2-18-induced programmed necrosis, HCC-1954 cells were incubated with the ROS scavenger N-acetylcysteine (NAC) or JNK inhibitor SP600125 before H2-18 treatment. As demonstrated in Figure 4C, the cell death caused by H2-18 was significantly reduced by prior exposure to both SP600125 and NAC.

### H2-18 increases programmed necrosis through transcriptional level

**Figure 5:**
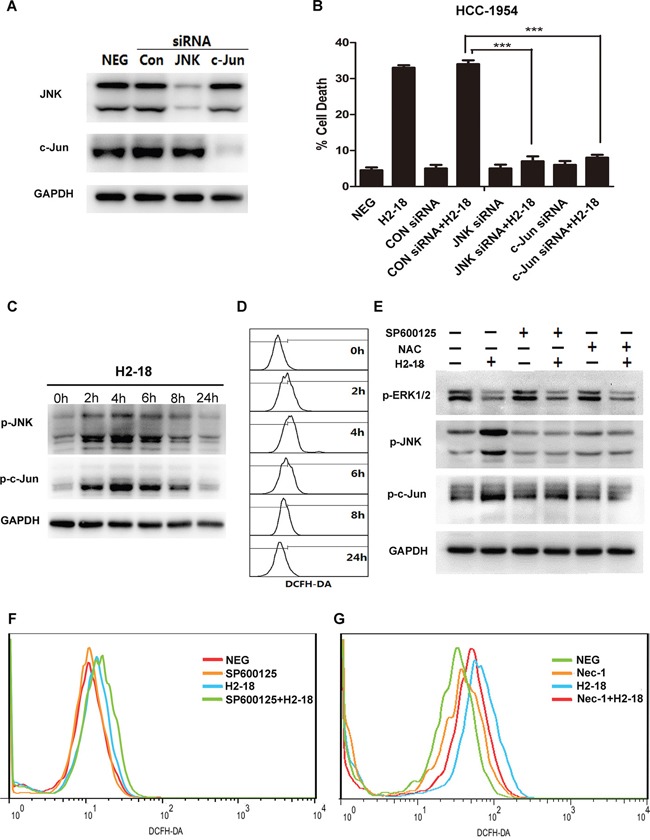
RIP1-ROS-JNK-c-Jun signaling pathway is involved in H2-18-induced programmed cell death **A.** Immunoblots determining knockdown of JNK or c-Jun by siRNA in HCC-1954 cells. **B.** The effects of knockdown of JNK or c-Jun on H2-18-induced cell death in HCC-1954 cells. Data are shown as means ± SD. *, *P*<0.05; **, *P*<0.01; ***, *P*<0.001; ANOVA. **C.** Immunoblots examining the level of pJNK and p-c-Jun in HCC-1954 cells treated with 10 μg/ml H2-18 at indicated time. **D.** DCFH-DA was detected by flow cytometry to measure the level of ROS production at indicated time in H2-18-treated HCC-1954 cells. **E.** Immunoblots detecting the effects of JNK inhibitor SP600125 or ROS scavenger NAC on pERK1/2, pJNK and p-c-Jun in H2-18-treated HCC-1954 cells. **F.** DCFH-DA was detected by flow cytometry to measure the level of ROS production in HCC-1954 cells treated with control IgG, JNK inhibitor SP600125, H2-18, and SP600125 plus H2-18. **G.** DCFH-DA was detected by flow cytometry to measure the level of ROS production in HCC-1954 cells treated with control IgG, necrosis inhibitor Nec-1, H2-18, and Nec-1 plus H2-18. Every experiment was repeated 3 times.

It is reported that JNK could drive cell death via two distinct pathways: impairing mitochondrial function by Bcl-2 family and regulating gene expression through c-Jun [29]. Our previous data showed that both Bcl-2 family and mitochondrial membrane potential were not changed by H2-18 (Figure 3C, 4D), whereas c-Jun was markedly activated by H2-18 (Figure 4A). To further examine the role of c-Jun in cell death, we knocked down c-Jun expression using a specific small-interfering RNA (siRNA). In HCC-1954 cells, both siRNA knockdown of c-Jun and JNK abolished H2-18-induced cell death (Figure 5A, 5B). Collectively, our data indicated that activation of JNK/c-Jun pathway was essential for H2-18-induced programmed necrosis.

### ROS is upstream of JNK in H2-18-induced cell death

Next, HCC-1954 cells treated with H2-18 for indicated time were lysed for western blotting analysis. An increase in the phosphorylation of both JNK and c-Jun was observed within the first 4h after H2-18 treatment, followed by a nearly 20-h recovery period (Figure 5C). Similarly, the level of ROS increased, reached a peak at around 4h with H2-18 treatment, and after that started to decrease (Figure 5D). To investigate the relationship between ROS and JNK, the ROS scavenger N-acetylcysteine (NAC) and JNK inhibitor SP600125 were used. The results showed that NAC treatment apparently attenuated H2-18-induced phosphorylation of JNK and c-Jun (Figure 5E). And the inhibition of JNK activation with SP600125 did not decrease H2-18-induced ROS production (Figure 5F).

### Activation of RIP1 leads to ROS production in H2-18-induced cell death

Since recent studies reported that activation of RIP1 could lead to ROS generation [30–33], we speculate that ROS is downstream of RIP1 in H2-18-induced cell death. This hypothesis was confirmed by the observation that the RIP1 inhibitor necrostatin-1 could inhibit H2-18-induced ROS production (Figure 5G). Compared with HCC-1954 cells treated with H2-18 alone, treatment with H2-18 plus necrostatin-1 produced significantly less ROS (Figure 5G).

### H2-18 potently inhibits the growth of trastuzumab-resistant tumors *in vivo*

**Figure 6:**
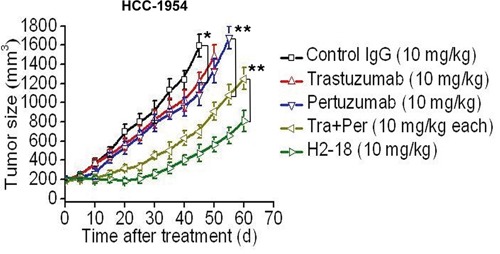
H2-18 potently inhibits the growth of trastuzumab-resistant tumors *in vivo* Tumor volume of HCC-1954 breast tumor xenografts after treatment with control IgG (10 mg/kg, twice a week, intravenously), trastuzumab (10 mg/kg, twice a week, intravenously), pertuzumab (10 mg/kg, twice a week, intravenously), trastuzumab plus pertuzumab (10 mg/kg for each, twice a week, intravenously) and H2-18 (10 mg/kg, twice a week, intravenously). Data are shown as means ± SEM. *, *P*<0.05; **, *P*<0.01; Mann-Whitney test.

The therapeutic efficacy of trastuzumab, pertuzumab, trastuzumab plus pertuzumab, and H2-18 was examined in nude mice bearing established HCC-1954 xenograft tumors. Compared with the control human IgG, both trastuzumab and pertuzumab could suppress the growth of HCC-1954 tumors (Figure 6). Trastuzumab plus pertuzumab was more efficient in inhibition of HCC-1954 tumors than either of the two mAbs alone (Figure 6). Importantly, H2-18 inhibited tumor growth much more effectively than the combination of trastuzumab and pertuzumab (Figure 6).

## DISCUSSION

It is well known that the ability of trastuzumab to inhibit the *in vitro* cell proliferation correlates with ErbB3/PI3K/AKT pathway inhibition [34]. For example, in trastuzumab-sensitive BT-474 cells, trastuzumab efficiently suppressed the cell proliferation through potent inhibition of pErbB3 and its downstream signaling molecule, pAkt [21]. However, trastuzumab was ineffective at inhibiting the growth of trastuzumab-resistant breast cell lines including HCC-1954. Although trastuzumab could reduce ErbB3 phosphorylation in HCC-1954 cells, it could not decrease pAkt. Compared with BT-474 cell line, HCC-1954 harbors an activating PIK3CA mutation (H1047R). Mutations in PIK3CA, a gene encoding the catalytic p110a subunit, were found in 30% of breast cancer [35]. Single amino acid substitution: E542K, E545K, or H1047R was responsible for 80% of the cancer-specific mutations in PIK3CA [35]. These “hot spot mutations” enhance the activity of the kinase, transform cells, and are oncogenic *in vivo* [11]. They uncouple PI3K activity from the ErbB2-ErbB3 oncogenic unit, resulting in PI3K/AKT pathway aberrant activation. This sustainably activated pathway in turn help tumor cells escape the action of trastuzumab and confer trastuzumab resistance. In the present study, trastuzumab, even trastuzumab in combination with pertuzumab, was unable to effectively inhibit the growth of trastuzumab-resistant cell line HCC-1954. However, H2-18 showed a significant antitumor effect on HCC-1954 cells, both *in vitro* and *in vivo*, suggesting that it may overcome trastuzumab resistance.

Next, we asked why H2-18 could circumvent the resistance to trastuzumab. Even when cancers progress after multiple ErbB2-directed therapies, ErbB2 still remains a valid therapeutic target [26, 27, 36, 37]. Thus, for some, most, or all ErbB2-positive cancers, ErbB2 itself continues to represent a major vulnerability. The challenge is to determine the optimal method to capitalize on this vulnerability [38]. H2-18, trastuzumab and pertuzumab binds different domains of ErbB2. Antibodies against distinct regions of ErbB2 may exert different functions. In previous studies, 7C2 and 7F3, anti-ErbB2 antibodies specific for domain I, have been demonstrated to induce PCD, but the involving mechanisms are not yet elucidated [36]. Moreover, the antitumor activity of 7C2 and 7F3 was not investigated in trastuzumab-resistant breast cancer cells [36]. Our present study indicated that H2-18 could induce PCD potently not only in trastuzumab-sensitive breast cancer cells, but also in trastuzumab-resistant breast cancer cells. More importantly, H2-18 effectively suppressed the *in vivo* growth of trastuzuamb-resistant breast cancer cell lines. Since HCC-1954 harbors aberrant activated PI3K/AKT pathway, the *in vivo* therapeutic efficacy of H2-18 in the trastuzumab-resistant cell line may be attributable to its markedly enhanced cell death-inducing activity. PCD is a cell suicide event, executed by delicately regulated mechanisms [37–39]. Beside apoptosis, programmed necrosis (necroptosis) also belongs to PCD [40, 41]. Induction of necroptosis could be a good antitumor strategy, even in many cases that cancer cells are resistant to current therapy [42]. It is well established that the involvement of receptor interaction protein kinase 1 and 3 (RIP1 and RIP3), JNK pathway and ROS are important in programmed necroptosis [37, 43, 44]. Our study has revealed that not PI3K/AKT pathway, but RIP1-ROS-JNK-c-Jun signaling pathway contributed to H2-18-induced cell death.

**Figure 7:**
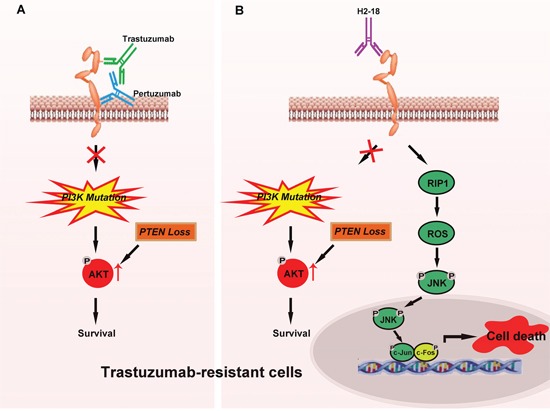
Model of H2-18 as an effective antibody conquering trastuzumab resistance by inducing programmed cell death **A.** In trastuzumab-resistant breast cancer cells, trastuzumab plus pertuzumab is unable to effectively suppress cell growth both *in vitro* and *in vivo* due to sustained activated PI3K/AKT signaling caused by PI3K mutation or PTEN loss in these cells. **B.** In trastuzumab-resistant breast cancer cells, H2-18 also fails to suppress the aberrant activated PI3K/AKT signaling caused by PI3K mutation or PTEN loss. However, H2-18 can overcome trastuzumab resistance *in vivo* possibly due to its potent ability to induce cell death. H2-18 treatment can activate RIP1, resulting in ROS production and then activation of the pro-necrotic effect of JNK, ultimately leading to programmed necrosis.

In trastuzumab-sensitive breast cancer cells, both trastuzumab and pertuzumab block ErbB2 dimerization and inhibit the activation of the main downstream pathways of ErbB2: PI3K/AKT and MAPK/ERK signaling pathways [7, 8, 17]. In trastuzumab-resistant breast cancer cells with PI3K mutation or PTEN loss, due to the persistently activated PI3K/AKT signaling, trastuzumab plus pertuzumab is unable to effectively suppress cell growth both *in vitro* and *in vivo* (Figure 7A). H2-18 also fails to suppress the aberrantly activated PI3K/AKT signaling in these cells (Figure 7B). However, H2-18 can exhibit great antitumor activity in trastuzumab-resistant cells *in vivo*. We conclude that this may be attributable to its potent ability to induce PCD (Figure 7B). H2-18 treatment activates RIP1, which leads to ROS production. Then the pro-necrotic effect of JNK was activated by ROS, ultimately resulting in programmed necrosis (Figure 7B). As some human breast cancers show decreased expression of RIP1 and RIP3, whether the expression of RIP1 and RIP3 in the breast cancer cells would affect the clinical effects of H2-18 still remains unclear. Also, the effect of H2-18 on primary breast tumors needs to be determined in the future studies.

In conclusion, our present study defines a new ErbB2 domain I-specific fully human antibody, H2-18. It shows a unique ability to induce PCD and more importantly, it can effectively circumvent the resistance to trastuzumab. These data offer interesting mechanistic insight into ErbB2 receptor structure and function. The H2-18 antibody might be a promising agent for treating ErbB2-overexpressing breast cancer.

## MATERIALS AND METHODS

### Cell lines and animals

The human breast cancer cell lines BT-474, SKBR-3, HCC-1954, HCC-1419, MDA-MB-231, and MCF-7 were obtained from the American Type Culture Collection. All the cell lines were authenticated twice by morphologic and isoenzyme analyses during the study period. Cell lines were routinely checked for contamination by mycoplasma using Hoechst staining and consistently found to be negative. Six-week-old female BALB/c nude mice were obtained from the Shanghai Experimental Animal Center of Chinese Academy of Sciences. All animals were treated in accordance with guidelines of the Committee on Animals of the Second Military Medical University.

### Phage display libraries and selections

Nonimmunized human single-chain variable fragment (scFv) phage display libraries were used for lead-scFv isolation. ErbB2-ECD, at 10 mg/ml in phosphate buffered saline (PBS), was immobilized on immunotubes (Nunc), and ErbB2-binding phage was isolated by 3 sequential rounds of panning [45]. The optimized variants were isolated by selection from randomized libraries in solution using biotinylated ErbB2 captured on streptavidin-coated paramagnetic beads (Dynal) [46]. The VH CDR3 randomized repertoires were constructed by polymerase chain reaction (PCR) using mutagenic oligonucleotides to replace the last 6 VH CDR3 amino acids with randomized codons. The mutated DNA was ligated into the phagemid vector pCANTAB6 and electroporated into *Escherichia coli* TG1 [47]. Libraries of 6 × 10^8^ individual clones were generated. The randomized libraries were subjected to 1 round of panning on 10 mg/ml of immobilized ErbB2-ECD, followed by 12 rounds of soluble selection using decreasing concentrations of biotinylated ErbB2-ECD, from 50 nM down to 100pM.

### Competitive binding assay

Cells at 1 × 10^6^ cells/mL were incubated with a subsaturating concentration of the indicated Alexa Fluor 488-conjugated anti-ErbB2 mAbs and increasing concentrations of purified competing antibodies for 1 hour at 4°C. Then, the cells were washed and analyzed by flow cytometry using a FACSCalibur (Becton Dickinson). The IC50 values of competitors were calculated using a four-variable algorithm.

### Cell viability assay

Cells were seeded at a density of 5 × 10^3^ cells per well in a flat-bottomed 96-well plate in humidified 37°C and 5% CO_2_ atmosphere. After 12 hours, the cells were treated with 10μg/ml control IgG, trastuzumab, pertuzumab, trastuzumab plus pertuzumab, and H2-18 for an additional 5 days. Cell viability was determined by CellTiter 96 AQueous One Solution Cell Proliferation Assay (MTS assay) Kit (Promega).

### Immunoblotting

Cells were treated with 5μg/ml anti-ErbB2 antibodies for 4h at 37°C. After washing, the cells were lysed in SDS lysis buffer and the cell lysates were subjected to SDS-PAGE and immunoblotted with antibodies against ErbB2, phospho-ErbB2-Tyr1221/1222, ErbB3, phospho-ErbB3-Tyr1289, AKT, phospho-AKT-Ser473, p44/42 MAPK, phospho-p44/42 MAPK-Thr202/Tyr204, SAPK/JNK, phospho-SAPK/JNK-Thr183/Tyr185, c-Jun, phospho-c-Jun-Ser63, PARP, Caspase-3, LC3A/B, Bak, Bax, Puma, Bid, Bim, Mcl-1, Bcl-xl, phospho-Bcl-2-Ser70, Bcl-2 (all from Cell Signaling Technology).

### Cell death assay

Cells were seeded in flat-bottomed 24-well plate at a density of 1 × 10^5^ cells per well in the growth medium and grown overnight at 37°C in a humidified incubator with 5% CO_2_. Cells were treated with 10μg/ml indicated anti-ErbB2 mAbs and IgG control for 24 h. Cell death was measured by using Dead Cell Apoptosis Kit with Annexin V Alexa Fluor® 488 & Propidium Iodide (PI) (Life Technologies) according to the manufacturer's protocol. Briefly, cells were harvested, washed once with binding buffer and resuspended in binding buffer at a cell density of 1 × 10^6^ cells/ml. 5 microlitres of 488-conjugated Annexin-V and 0.1 microlitres of PI were added to 100 μl of the cell suspension and incubated for 15 min at room temperature. Then, the cells were washed with binding buffer and resuspended in 200 μl binding buffer, analyzed by flow cytometry on a FACSCalibur (Becton Dickinson).

### Inhibitor assay

For the inhibitor assay, overnight grown HCC-1954 cells were first treated with 10 μM pan caspase inhibitor Z-VAD-FMK (Calbiochem), or 100 nM autophagy inhibitor bafilomycin A1 (Sigma), or 5 μM necrosis inhibitor necrostatin-1 (Nec-1, Calbiochem) for 2 h before co-treated with H2-18. After 24 h incubation at 37°C, cell death assay was carried out as described in previous section. To study the effect of MAPKs and ROS on H2-18-induced cell death in HCC-1954 cells, 5 μM SP600125 (a specific JNK inhibitor, Sigma) or 5 mM NAC (a ROS scavenger, Sigma) was added to cells for 2 h prior to H2-18 co-treatment. Cell death assay and Western blot were performed as described in the previous section.

### Transmission electron microscope analysis

Cells were seeded in a 100 mm dish and treated with indicated anti-ErbB2 mAbs and IgG control for 48 hours. Then cultured cells were washed twice with PBS, harvested by cell scraper, and then fixed for 12 h in 2.5% glutaraldehyde in 30 mM HEPES buffer at 4°C. And the samples were treated with 1.5% osmium tetroxide, dehydrated with acetone and embedded in durcupan resin. Thin sections were post-stained with lead citrate and examined in the Hitachi H-7650 transmission electron microscope at 100 kV.

### Mitochondrial membrane potential assay

Mitochondrial membrane potential (MMP) was probed by MITOSCREEN JC-1 KIT (BD Biosciences) according to the manufacturer's instruction. JC-1 staining is detected by flow cytometry on a FACSCalibur (Becton Dickinson). JC-1 is a dye that oligomerizes in normal mitochondria with red fluorescence. Loss of red fluorescence indicates loss of mitochondrial membrane potential.

### ROS detection

ROS detection was evaluated with 2′, 7′-dichlorofluorescin diacetate (DCFH-DA, Sigma) and dihydroethidium (DHE, Life Technologies). Cells were seeded at a density of 1 × 10^5^ cells per well in a flat-bottomed 24-well plate. After cells were treated with indicated anti-ErbB2 mAbs for 0-24 h, DCFH-DA or DHE was added to the cells (final concentration at 10μM and 5μM in PBS, respectively) and incubated for 20 min at 37°C. Extracellular DCFH-DA or DHE was then removed by washing the cells twice with PBS. The fluorescence of the cells loaded with DCFH-DA and DHE was measured with FACSCalibur (Becton Dickinson) using excitation wavelength of 485 nm and emission wavelength of 535 nm.

### Transfection of small interfering RNA (siRNA)

Transfection of siRNA was performed by using DharmaFECT 4 transfection reagent (Dharmacon) according to the manufacturer's instructions. The JNK siRNA (5′- GCC CAG UAA UAU AGU AGU ATT-3′), c-Jun siRNA(5′-ACG CAA ACC UCA GCA ACU UTT-3′), and control siRNA(5′-UUC UCC GAA CGU GUC ACG UTT-3′) were purchased from GeneParma Company (Suzhou, China). After siRNA transfection for 24 hour, the cells were incubated with or without H2-18 for subsequent experiments.

### *In vivo* therapy study

For xenograft studies, female BALB/c nude mice were implanted with HCC-1954 cells (5 × 10^6^ per mouse) in right mammary fat pad. When tumors reached an average tumor volume of 100-200 mm^3^, animals were distributed into treatment cohorts of 10 mice each randomly. Different anti-ErbB2 mAbs were injected intravenously twice weekly for 4 consecutive weeks. Control mice were administered vehicle (IgG) alone. Tumors were measured with digital calipers twice a week and tumor volumes were calculated by the formula: volume (mm^3^) =length × (width) ^2^/2.

### Statistical analysis

The data were tested for parametric distribution before applying parametric analysis. Statistical analysis was conducted by Student's unpaired *t* test or ANOVA to identify significant differences unless otherwise indicated. Differences were considered significant at *P* < 0.05.

## SUPPLEMENTARY MATERIAL FIGURES


