# Supplementary material for: An anti-ErbB2 fully human antibody circumvents trastuzumab resistance
Source: Oncotarget. 2016 Aug 24;7(41):67129–41. doi: 10.18632/oncotarget.11562 (PMC5341862; doi:10.18632/oncotarget.11562)
Supplement: Supplementary file 1 [file oncotarget-07-67129-s001.pdf]

An anti-ErbB2 fully human antibody circumvents trastuzumab resistance

SUPPLEMENTARY FIGURES

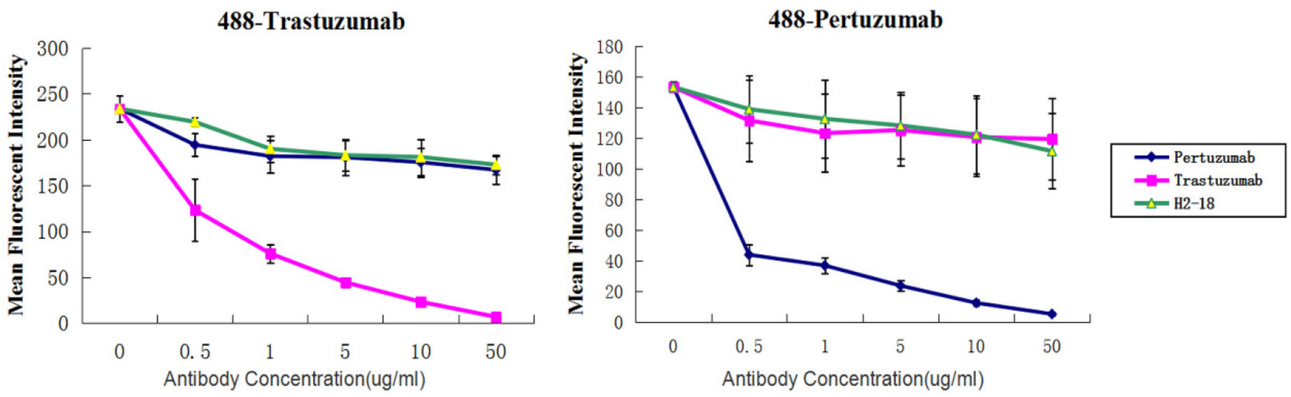

Supplementary Figure S1: Competitive binding assays. Trastuzumab, pertuzumab, and H2-18 were evaluated for their ability to compete with Alexa Fluor 488-labeled trastuzumab or Alexa Fluor 488-labeled pertuzumab for binding to ErbB2 in HCC-1954 cells.

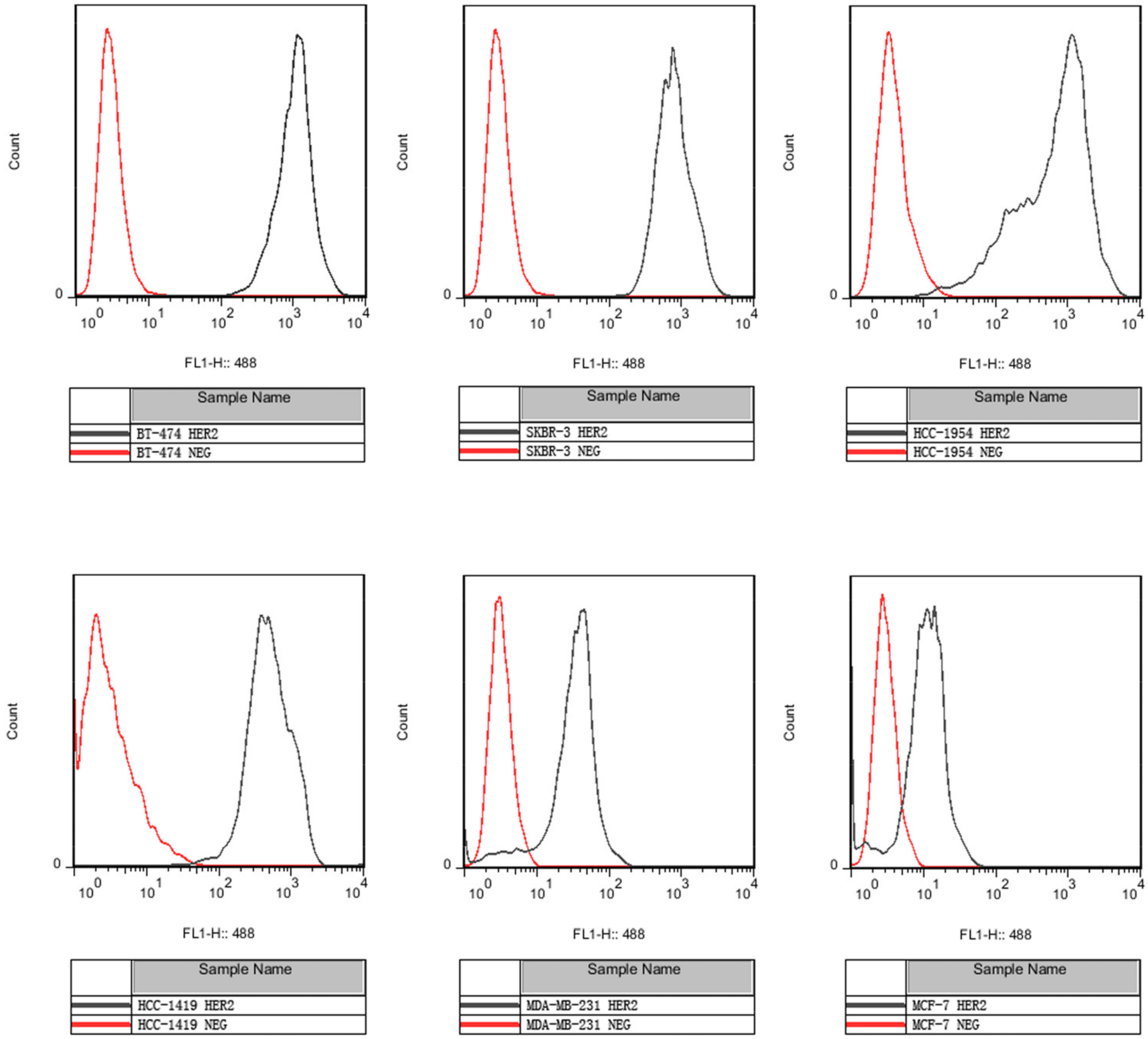

Supplementary Figure S2: The expression of ErbB2 in the human breast cancer cell lines BT-474, SKBR-3, HCC-1954, HCC-1419, MDA-MB-231 and MCF-7. 1×10^{6} cells were incubated with 100µl trastuzumab (1 mg/ml) on ice for 40 min. After washing with PBS, the cells were incubated with Alexa Fluor ® 488-labeled goat anti-human IgG (H+L) on ice for 30 min. Then, the cells were washed with PBS and resuspended in 200 µl binding buffer, analyzed by flow cytometry on a FACSCalibur (Becton Dickinson).

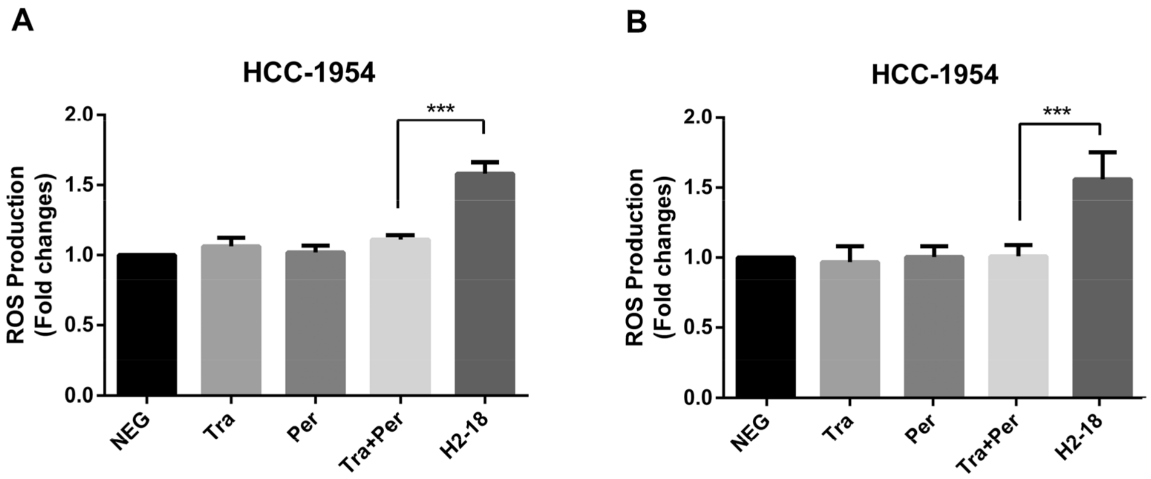

Supplementary Figure S3: ROS production induced by control IgG, trastuzumab, pertuzumab, trastuzumab plus pertuzumab, and H2-18. The HCC-1954 cells were treated with indicated mAbs and loaded with DCFH-DA or DHE. DCFH-DA (A) or DHE (B) was quantified and the fold changes against the control group were shown.

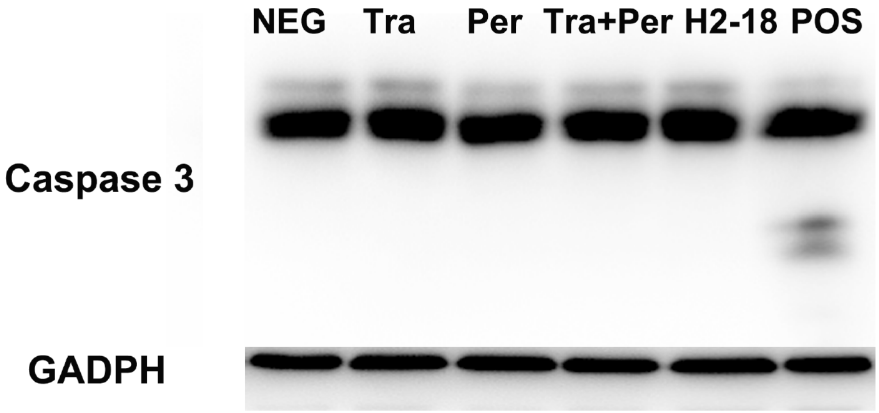

Supplementary Figure S4: Immunoblots evaluating the caspase 3 proteins of HCC-1954 cells treated with 10 µg/ml of control IgG, trastuzumab, pertuzumab, trastuzumab plus pertuzumab, and H2-18. Lysates of HCC-1954 cell treated with NaN3 were used as positive control.

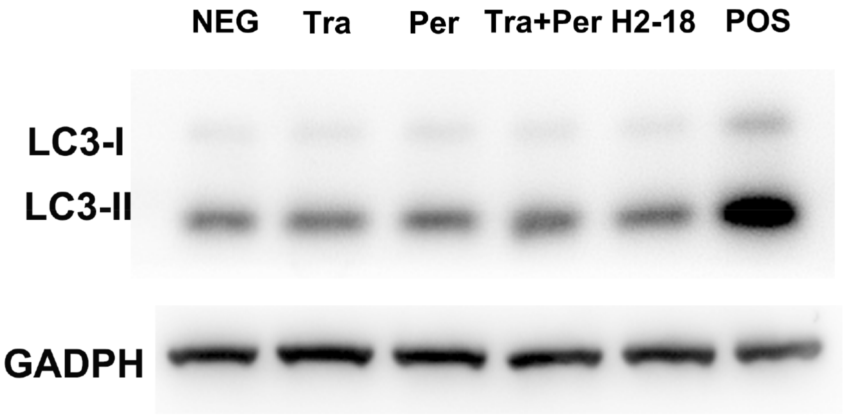

Supplementary Figure S5: Immunoblots evaluating the LC3-I and LC3-II proteins of HCC-1954 cells treated with 10 µg/ml of control IgG, trastuzumab, pertuzumab, trastuzumab plus pertuzumab, and H2-18. Lysates of cells treated with Earle's balanced salts solution (EBSS) for 6h were used as positive control. The presentative images out of three independent experiments were shown.
